# Trocar-assisted, flanged haptics, sutureless intrascleral fixated intraocular lens implantation combined with Descemet membrane endothelial keratoplasty

**DOI:** 10.5935/0004-2749.20200100

**Published:** 2024-02-11

**Authors:** Remzi Karadag

**Affiliations:** 1 Department of Ophthalmology, School of Medicine, Istanbul Medeniyet University, Goztepe, Istanbul, Turkey

**Keywords:** Descemet membrane, Keratoplasty, penetrating, Lens implantation, intraocular, Lenses, intraocular, Sclera/surgery, Humans, Case reports, Lâmina limitante posterior, Ceratoplastia penetrante, Implante de lente intraocular, Lentes intraoculares, Esclera/ cirurgia, Humanos, Relatos de casos

## Abstract

This article reports a combined technique of sutureless intrascleral fixated intraocular lens implantation and Descemet membrane endothelial keratoplasty in a patient with anterior pseudophakic bullous keratopathy. Two scleral tunnels were created, corneal incisions were made, and a foldable intraocular lens was cut and removed from the anterior chamber. After performing anterior vitrectomy, a 3-piece foldable intraocular lens was implanted into the anterior chamber. One of the intraocular lens haptics was grasped with a forceps and pulled out from the scleral tunnel. Then, the end of the haptic was cauterized. Similar maneuvers were applied for the other haptic. Next, an 8-mm-diameter donor tissue was prepared, and the recipient endothelial tissue was peeled and removed from the center of the recipient cornea. The prepared donor tissue was injected into the anterior chamber. After proper opening and placement of the donor tissue, an air bubble was injected below the tissue. There were no postoperative complications during the 1-month follow-up.

## Abstract (pt)

Relato de uma técnica que combina o implante de uma lente intraocular com fixação intraescleral sem sutura e uma ceratoplastia endotelial da membrana de Descemet em paciente com ceratopatia bolhosa pseudofácica anterior. Foram criados dois túneis esclerais. Foram feitas incisões na córnea e a lente intraocular dobrável foi cortada e removida da câmara anterior. Foi então efetuada uma vitrectomia anterior e uma lente intraocular dobrável de 3 peças foi implantada na câmara anterior. Um dos hápticos da lente intraocular foi pinçado com um fórceps e puxado para fora do túnel escleral. A extremidade do háptico foi cauterizada. Manobras semelhantes foram feitas no outro háptico. Foi preparado um tecido de doador com 8 mm de diâmetro e o tecido endotelial da área receptora foi removido do centro da córnea. O tecido preparado do doador foi injetado na câmara anterior. Após abertura e posicionamento adequados do tecido do doador, foi injetada uma bolha de ar abaixo do tecido. Não foi observada nenhuma complicação pós-operatória durante um mês de acompanhamento.

## INTRODUCTION

There are several combined methods for treating patients with insufficient capsular support and pseudophakic bullous keratopathy due to an anterior chamber (AC) intraocular lens^(1^-^6)^. Some of these methods include intraocular lens (IOL) implantation techniques such as iris-fixated IOL and scleral-fixated IOL implantation and corneal transplantation techniques such as penetrating keratoplasty, Descemet membrane endothelial keratoplasty (DMEK), and Descemet stripping endothelial keratoplasty^(1^-^6)^. DMEK is a new posterior lamellar keratoplasty method by which the surgeon can replace only the removed dysfunctional endothelial and Descemet membrane tissues with a new tissue without harming the other corneal tissues^(7)^.

Sutureless scleral-fixated (SSF) IOL implantation methods^(8)^ and DMEK^(7)^, which have recently become popular, are the newest methods in their own categories. Till date, only a few studies have been reported in the literature regarding SSF IOL implantation combined with keratoplasty techniques; however, these were only case reports^(1^-^5)^.

In this article, our aim was to describe the combined surgery of trocar-assisted SSF IOL implantation and DMEK at the same session in a patient with pseudophakic bullous keratopathy.

## CASE REPORT

**Figure 1:**
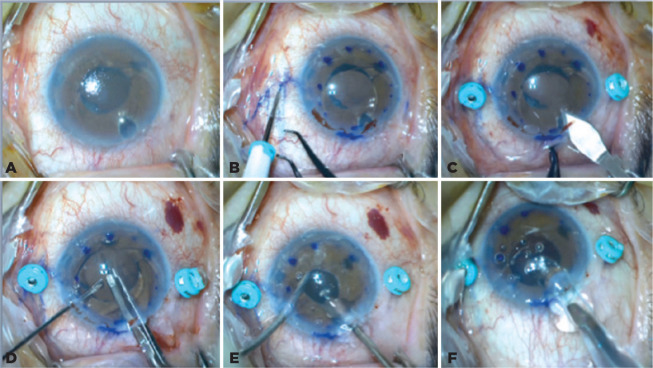
A) Pseudophakic bullous keratopathy caused due to the foldable anterior chamber IOL; B) Two 3-mm scleral tunnels are created 2 mm away from and parallel to the limbus with the 23-gauge vitrectomy trocars; C) Main corneal incision is performed; D) The foldable IOL in the anterior chamber is cut using an IOL scissor; E) Anterior vitrectomy is performed; F) A 3-piece foldable IOL is implanted into the anterior chamber with an injector.

A 67-year-old female patient who underwent complicated cataract surgery 13 months ago in another clinic had pseudophakic bullous keratopathy that was caused due to AC IOL implantation. Her visual acuity was defined by counting fingers at 4 m, and the intraocular pressure was 15 mmHg. A detailed slit-lamp examination revealed the presence of a foldable posterior chamber IOL in the AC (Figure 1A). Removal of the AC IOL, trocar-assisted SSF IOL implantation^(1^,^8)^, and DMEK surgeries were performed as explained briefly below. Edematous corneal epithelium was removed using a spatula. Two 3-mm scleral tunnels were created 2 mm away from and parallel to the limbus using 23-gauge vitrectomy trocars that entered the sclera transconjunctivally at the 3 O’clock and 9 O’clock meridians at an angle of approximately 10° (Figure 1B) and entered into the posterior chamber^(1^,^8)^. Corneal main and side port incisions were created using 3.0 (Figure 1C) and microvitreoretinal (MVR) knives, respectively. After injecting a viscoelastic device, we observed that one of the haptics of the IOL was attached to the AC angle on the temporal side. This haptic was detached from the AC angle using a sinskey hook. The foldable IOL was cut with IOL scissors (Figure 1D) and then removed from the main corneal incision using forceps.

**Figure 2:**
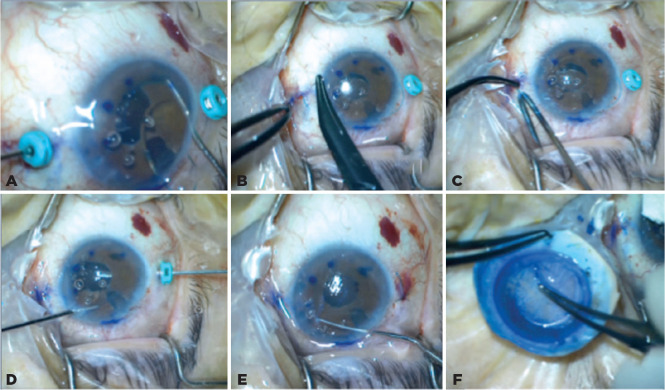
A) The tip of one of the IOL haptics is grasped using a 23-gauge serrated retinal forceps; B) A transconjunctival safety 10-0 nylon suture is placed at the scleral tunnel entry site; C) The end of the haptic is cauterized to make a flange; D) The tip of the other haptic is grasped using a forceps; E) Recipient endothelial tissue is peeled and removed from the center of the recipient cornea using a reverse sinkey hook; F) An 8-mm-diameter DMEK donor tissue is prepared.

**Figure 3:**
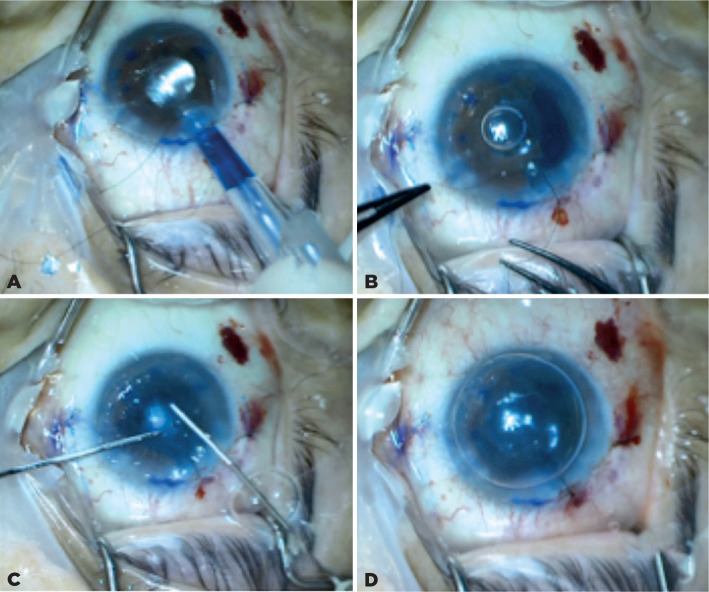
A) The DMEK donor tissue is delivered into the anterior chamber with an injector; B) The main corneal incision is closed using 10-0 nylon suture; C) Recipient cornea is constantly tapped from the epithelial side to unroll the graft in the swallowed anterior chamber; D) At the end of the surgery.

After performing triamcinolone-assisted anterior vitrectomy (Figure 1E), a posterior chamber 3-piece foldable IOL with propylene haptic was implanted into the AC with an injector through the main corneal incision (Figure 1F). The tip of one of the IOL haptics was grasped with a 23-gauge serrated retinal forceps and entered through the one of the trocar’s cannula (Figure 2A), and the haptic and the 23-gauge cannula were together pulled out from the scleral tunnel simultaneously. A transconjunctival safety 10-0 nylon suture, which was removed at the end of the first postoperative week, was placed (Figure 2B) at the scleral tunnel entry^(1^,^8)^. The end of the haptic was cauterized to make a flange (Figure 2C). Similar maneuvers were applied for the other haptic (Figure 2D). Intraoperative miosis was achieved by injecting 0.01% carbachol solution (Miostat, Alcon Laboratories Inc., San Diego, USA) into the AC. Next, an 8-mm-diameter DMEK donor tissue was prepared (Figure 2F), after which an 8-mm-diameter epithelial mark was made to the outline of the area of the Descemet membrane excision on the recipient cornea. Then, the recipient endothelial tissue was peeled and removed from the center of the recipient cornea using a reverse sinkey hook (Figure 2E). The prepared DMEK donor tissue was injected into the AC through the main corneal incision using an injector (Figure 3A), and the main corneal incision was closed using 10-0 nylon suture (Figure 3B). The recipient cornea was constantly tapped from the epithelial side to unroll the graft in the swallowed AC (Figure 3C).

After proper opening and placement of the donor tissue, an air bubble was injected below the tissue to ensure proper placement of the graft onto the recipient’s posterior stroma. Peripheral iridectomy was not performed because the recipient’s eye had already received this procedure. The surgery was completed without any complication (Figure 3D). In the postoperative course, topical dexamethasone, antibiotic eye drops, and artificial tear drops were initiated four times a day for 2 weeks. Afterward, the antibiotic eye drops and the artificial tear drops were stopped but the dexamethasone eye drops were continued twice a day for 2 weeks. In the first postoperative month, the cornea was clear and transparent, the intraocular pressure was 16 mmHg, and the visual acuity level of the eye was 0.7 decimal due to macular complications associated with age-related macular degeneration. Furthermore, there were no postoperative complications during the 1-month follow-up.

## DISCUSSION

There is controversy regarding the most appropriate technique of secondary IOL implantation during keratoplasty in an eye with insufficient capsular support^(1^-^7)^. Furthermore, this situation becomes more important in endothelial keratoplasty procedures because of the requirement of iris lens or iris IOL diaphragm^(7)^. In general, surgeons prefer iris-fixated or scleral-fixated IOLs rather than AC IOL implantation because of the nearness to the corneal endothelium^(8)^. Some surgeons prefer suture or SSF IOL implantation because this is the closest to the location of natural lens. The IOL does not compromise the corneal endothelium in both scleral-fixated IOL implantation techniques. Recently, there has been an increasing use of the SSF IOL implantation technique as it does not cause suture-related complications. In addition, as a scleral flap is not created, the duration of surgery has also become shorter^(8)^.

As the first combined surgery, Prakash et al. described a fibrin glue-assisted sutureless scleral fixation technique with a femtosecond laser-assisted penetrating keratoplasty surgery in three cases and did not observe any IOL-related and keratoplasty-related complications^(2)^. Another study similarly reported that there were no complications with fibrin glue-assisted intrascleral fixation combined with Descemet stripping automated endothelial keratoplasty or penetrating keratoplasty^(4)^. In our previous study, we described about the trocar-assisted intra-scleral fixated IOL implantation combined with penetrating keratoplasty in four patients^(1)^, wherein we did not observe any complications. Later, the implantation of SSF IOL using a 26-gage needle with penetrating keratoplasty combined surgery in 10 cases was reported by Sethi et al.^(9)^ who also did not find any intraoperative complications. In addition to these combined surgeries, Jacob et al. reported a combined techni que of DMEK with glued intrascleral haptic fixation of a posterior chamber IOL^(7)^. Their technique was the first combined method of DMEK and fibrin glue-assisted intrascleral fixated IOL^(7)^.

In the present case, DMEK and trocar-assisted SSF IOL implantation combined surgery was performed in a patient with anterior pseudophakic bullous keratopathy. To the best of our knowledge, this is the first case report of a patient in the literature who has undergone surgery through this method. Hypotony is one of the potential complications associated with SSF IOL implantation^(8^,^10)^. It may occur with combined SSF IOL implantation and DMEK and can cause subsequent graft detachment in the postoperative period. In our technique, this risk was prevented using transconjunctival secure suture, which was removed at the end of the first postoperative week. This combined procedure also permits a stable AC.

In conclusion, the trocar-assisted SSF method is a suitable surgical procedure for combination with endothelial keratoplasty as it requires few intraocular manipulations and provides iris IOL diagrams.

## References

[1] Karadag R, Bayramlar H, Azari AA, Rapuano CJ. (2016). Trocar-assisted, sutureless, scleral-fixated intraocular lens
implantation combined with penetrating keratoplasty. Cornea.

[2] Prakash G, Jacob S, Ashok Kumar D, Narsimhan S, Agarwal A, Agarwal A. (2009). Femtosecond-assisted keratoplasty with fibrin glue-assisted
sutureless posterior chamber lens implantation: new triple
procedure. J Cataract Refract Surg.

[3] Bhandari V, Reddy JK, Siddharthan KS, Singhania N. (2016). Simultaneous Descemet’s membrane endothelial keratoplasty and
posterior iris-claw-fixated intra ocular lens implantation (IOL) in
management of aphakic bullous keratopathy. Int Ophthalmol.

[4] Sinha R, Shekhar H, Sharma N, Tandon R, Titiyal JS, Vajpayee RB. (2012). Intrascleral fibrin glue intraocular lens fixation combined with
Descemet-stripping automated endothelial keratoplasty or penetrating
keratoplasty. J Cataract Refract Surg.

[5] Narang P, Agarwal A, Dua HS, Kumar DA, Jacob S, Agarwal A. (2015). Glued intrascleral fixation of intraocular lens with
pupilloplasty and pre-descemet endothelial keratoplasty: a triple
procedure. Cornea.

[6] Cervantes LJ. (2017). Combined double-needle flanged-haptic intrascleral fixation of an
intraocular lens and Descemet-stripping endothelial
keratoplasty. J Cataract Refract Surg.

[7] Jacob S, Agarwal A, Kumar DA, Agarwal A, Agarwal A, Satish K. (2014). Modified technique for combining DMEK with glued intrascleral
haptic fixation of a posterior chamber IOL as a single-stage
procedure. J Refract Surg.

[8] Karadag R, Celik HU, Bayramlar H, Rapuano CJ. (2016). Sutureless intrascleral fixated intraocular lens
implantation. J Refract Surg.

[9] Sethi HS, Naik MP, Gupta VS. (2016). 26-G needle-assisted sutureless glueless intrascleral haptic
fixation for secondary ciliary sulcus implantation of three-piece
polymethylmethacrylate intraocular lens during penetrating
keratoplasty. Taiwan J Ophthalmol.

[10] Totan Y, Karadag R. (2013). Two techniques for sutureless intrascleral posterior chamber IOL
fixation. J Refract Surg.

